# Surgical treatment of extensive hepatic alveolar echinococcosis using a three-dimensional visualization technique combined with allograft blood vessels: A case report

**DOI:** 10.1097/MD.0000000000021336

**Published:** 2020-07-31

**Authors:** Tiezheng Wang, Guangming Li, Zhi Fu, Daming Gao, Ning Li, Dongdong Lin

**Affiliations:** a Department of General Surgery, Beijing YouAn Hospital, Capital Medical University; b Beijing Institute of Hepatology, Beijing, China.

**Keywords:** hepatic alveolar echinococcosis, reconstruction, stenosis, surgical treatment, three-dimensional visualization technique

## Abstract

**Rationale:**

Hepatic alveolar echinococcosis (HAE) presents a high pathogenicity and case fatality rate. The main treatment for HAE is surgical resection. Giant lesions in the liver and invasion of the pathogen into the retrohepatic inferior vena cava are usually associated with a poor prognosis when radical resection cannot be performed.

**Patient concerns:**

A 56-year-old man who underwent hydatidectomy 7 years prior noted a recurrence of HAE. He was subsidized and admitted to our hospital for the purpose of surgical treatment.

**Diagnosis:**

By computed tomography, angiography and three-dimensional (3D) computed tomography reconstruction images, multiple, giant HAE with 75% stenosis was confirmed.

**Interventions:**

With the 3D visualization technique, we designed the surgical plan and performed radical resection of the lesions, including the invaded inferior vena cava, and maximized retention of normal liver tissue. The abdominal aorta of an organ donor was used for vascular allograft reconstruction.

**Outcomes:**

The patient recovered gradually after the operation. He was followed up for 3 months, and the reconstructed vein patency was good.

**Lessons:**

The 3D visualization technique combined with a blood vessel allograft allowed us to expand indications for radical resection of extensive HAE.

## 1 Introduction

Echinococcus multilocularis is a potential lethal pathogen causing hepatic alveolar echinococcosis (HAE), which presents a high pathogenicity and case fatality rate, especially in Asia.^[1]^ At present, radical resection is the preferred treatment for HAE.^[2,3]^ Patients with multiple giant HAE usually have a poor prognosis if radical resection cannot be performed due to low future remnant liver volumes.^[4]^ Furthermore, the invasion of the retrohepatic inferior vena cava presents a large barrier to successful treatment.^[2,5]^ In such cases, a three-dimensional (3D) visualization system is crucially needed^[6]^ for surgeons to better visualize difficult anatomy and improve patient safety .^[7,8]^ In this report, we describe a case of multiple and giant hepatic lesions with 75% stenosis of the inferior vena cava, which is usually considered to be incurable. Via the 3D visualization technique, we resected the lesion and successfully reconstructed the inferior vena cava with allograft blood vessels.

## 2 Case presentation

### 2.1 Preoperative evaluation

A 56-year-old man who underwent hydatidectomy 7 years prior noted a recurrence of HAE 1 year ago. The patient had no history of hepatitis or heavy drinking. He was a veterinarian and had lived in Tibet, China, all the time. Moreover, there was no family medical history related to the illness. The patient only had mild pain without fever, jaundice, or other symptoms. He was admitted by us with the suspicion of HAE.

**Figure 1:**
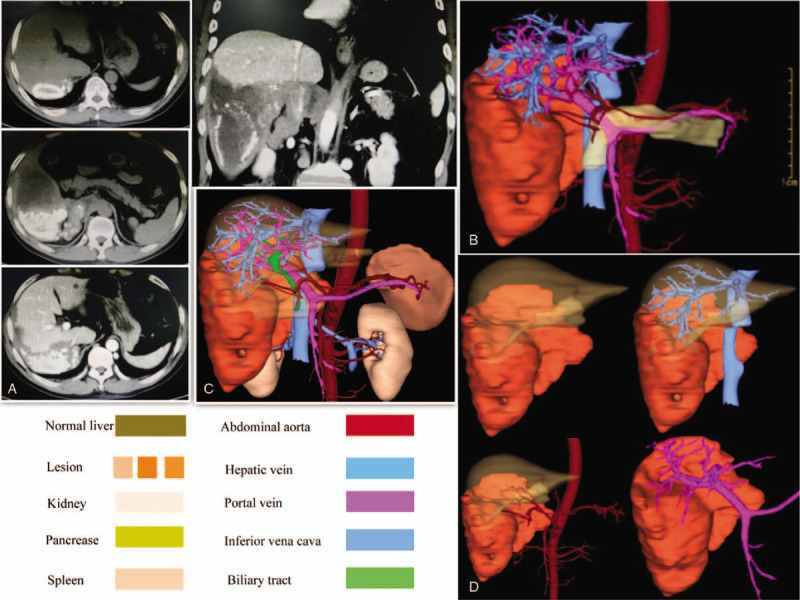
Wide range of invasive liver segments (I, II, V, VI, VIII, and IX) and suspected invasion of the right renal pedicle (A). Digital organ model image reconstructed by 3D visualization technique: position, size, number of liver lesions; inflow, outflow and biliary tract of the liver (B-D).

**Figure 2:**
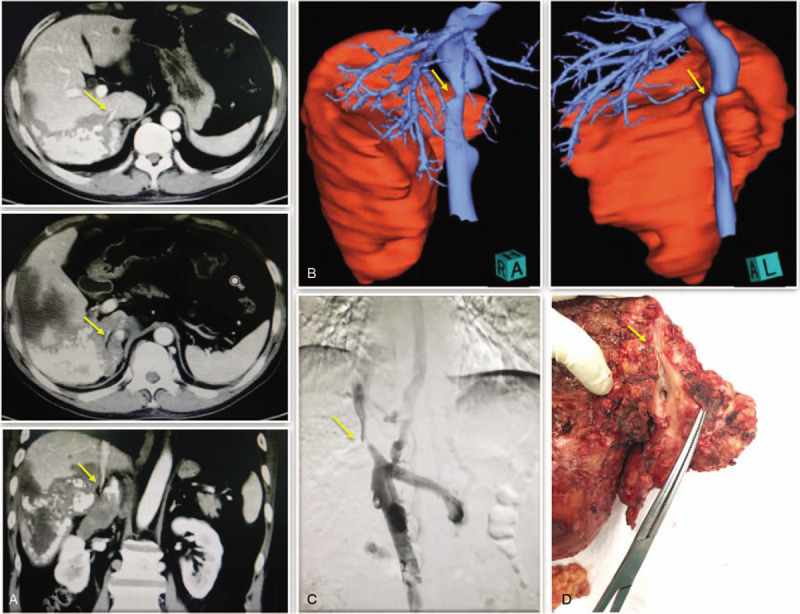
Severe stenosis of RHSIVC, eroded by the giant lesion, was detected by CT (A), 3D-reconstructed model (B) and vena cava angiography (C) preoperatively and confirmed by pathological specimens postoperatively (D) (marked with yellow arrow).

**Figure 3:**
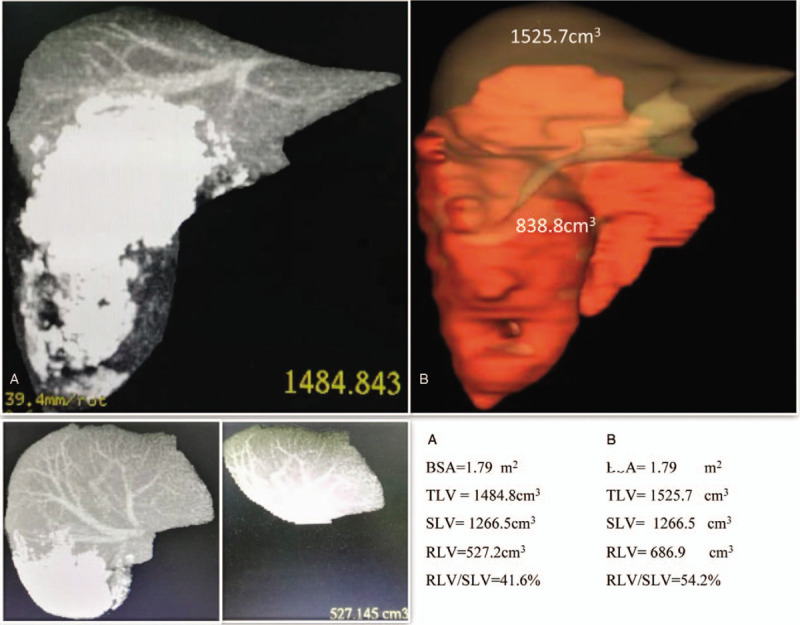
Assessment of resectability and compensation ability of residual liver by CT three-dimensional image postprocessing (A) and 3D visualization technique (B).

The basic characteristics of the patient were as follows: height = 170 cm, weight = 71 kg, and body mass index = 24.6. Both laboratory inspection and functions of vital organs showed no abnormality, except for slight elevations of transaminase (alanine aminotransferase 111.8 U/L and aspartate aminotransferase 61.1 U/L; normal range 9–50 U/L). However, abdominal computed tomography showed a giant hepatic lesion (20 cm in diameter) (Fig. 1). Lesions were mainly located on the right liver due to exogenous growth. Some parts of the lesion appeared less dense than the surrounding liver tissue, and necrosis of the damaged tissue was suspected. Right portal vein embolization and right adrenal gland invasion were observed. The lesions were characterized by widely invasive segments (I, II, V, VI, VIII, and IX). The retrohepatic segment of inferior vena cava (RHSIVC) was also invaded, leading to severe stenosis (Fig. 2). Inferior vena cava angiography was subsequently conducted to evaluate the degree of stenosis (Fig. 2C). computed tomography data were finally imported into 3D visualization software for preoperative assessment. Eventually, multiple, giant hepatic lesions with 75% stenosis were confirmed. We calculated the total/residual liver volumes based on the reconstructed 3D images using the computing function of the workstation and computed tomography 3D image postprocessing as a control. For this patient, body surface area = 1.79 m^2^; total liver volume = 1525.7 cm^3^, standard liver volume (SLV) = 1266.5 cm^3^ and RLV/SLV = 54.2%. These data were determined based on the 3D visualization technique. For the control, total liver volume = 1484.8 cm^3^, SLV = 1266.5 cm^3^ and RLV/SLV = 41.6%. We concluded that residual normal liver tissue can afford hepatectomy (Fig. 3).

### 2.2 Intraoperative results

**Figure 4:**
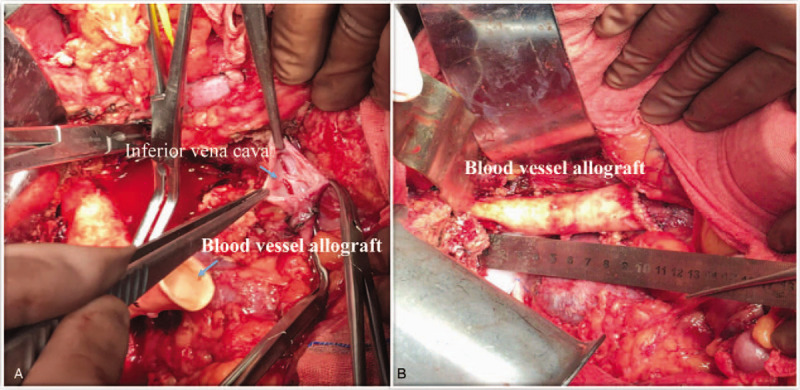
Inferior vena cava reconstruction using allograft abdominal aorta (a, b. AAA: abdominal aorta allograft; IVC: inferior vena cava, marked with white arrows).

Hepatectomy was carried out according to the preoperative reconstructed 3D model. Intraoperative findings were consistent with preoperative 3D visualization results: Multiple, giant lesions in the liver were confirmed. The lesions surrounded the RHSIVC to the right border of the abdominal aorta. Lesions of the RHSIVC were approximately 10 cm in length and could not be completely preserved. Invasion of the capsule and anterior pedicle of the right kidney was also found (Figs. 1 and 2). We performed irregular resection along the edge of lesions for radical resection of the lesion and maximal retention of normal liver tissue. The lesions were removed together with the invaded vena cava (Fig. 2D). Then, we chose a section of abdominal aorta from an organ donor on the same day to use as a blood vessel allograft. The allograft was 2 cm in diameter and 10 cm in length with a 4–0 prolene continuous eversion suture (Fig. 4). Air embolisms occurred twice during vena cava reconstruction. The first air embolism occurred because the proximal cardiac venous occlusion forceps did not grasp tightly. The second one was due to the application of argon spray to a liver section to stop bleeding. Both embolisms caused low blood pressure and fast heart rate (ABP 36/30 mm Hg, HR 142 bpm), and the vital signs recovered smoothly after the active rescue. In total, the surgery lasted for 11 hours. The patient was transfused with 20 units of erythrocyte suspension and 1200 mL of fresh-frozen plasma. Blood loss was approximately 3500 mL.

### 2.3 Postoperative and follow-up results

After the operation, the patient was transferred to the surgical intensive care unit, and the postoperative course was uneventful. The patient was discharged on the 27th day after the operation. The final pathology report reconfirmed the diagnosis of large HAE and vena cava stenosis (Fig. 3). Albendazole (20 mg/kg/d) was administered 3 days preoperatively and planned to continue for 1 year postoperatively.^[9]^ He was followed up for 3 months, and the blood vessel allograft showed good patency.

## 3 Discussion

HAE is a rare but potentially lethal parasitic disease.^[5,10]^ At present, radical resection is regarded as the preferred treatment for HAE.^[2,3,11]^ Due to the long clinical latency, many patients are not diagnosed until progressing to the late stage of the disease, when the lesions exceed the resectable limits.^[12–14]^ In this case, the giant hepatic lesion (20 cm in diameter), characterized by extensive involvement of liver segments (I, II, V, VI, VIII, and IX) and 75% stenosis of RHSIVC, might cause the patient to miss the opportunity for surgery.

It is acknowledged that the success of liver surgery is largely determined by the preoperative accurate understanding of intrahepatic structure and liver volume due to the complexity of liver anatomy.^[5,7]^ The 3D visualization technique emerged and was first applied for the planning of liver resection by Oldhafer^[15]^ et al in 1999. With 3D visualization techniques, comprehensive anatomic information, such as the position, size, number of hepatic lesions and adjacent structures, can be intuitively assessed. Moreover, 3D visualization vividly reflects the structural characteristics of the intrahepatic biliary and vascular network. The remaining liver volume and the diameter of blood vessels in the section were calculated to evaluate the feasibility of the proposed surgical strategy (Figs. 1–3). Depending on the outcomes of the virtual surgery and calculation, we concluded that this patient was eligible for radical surgery of hydatid lesions and that it was necessary to remove and reconstruct the eroded RHSIVC. In line with our expectations, the surgical procedure was consistent with that designed in the preoperative virtual surgery. Therefore, we believe that the 3D visualization technique is especially useful for the preoperative assessment and surgical planning of hepatectomy for end-stage HAE patients and that it greatly improves the success rate and reduces the risks of surgery.^[7,8]^

In this case, the patient's RHSIVC was corroded by a hydatid lesion infringing on the right edge of the abdominal aorta and could not be separated. Angiography suggested that vena cava stenosis occurred and umbilical veins were opened and widened compensatorily. Resecting the RHSIVC without reconstruction may be a choice.^[2,5]^ Ligation is considered prohibitively risky unless in the context of trauma damage control.^[16]^ Compared with vena cava reconstruction/repair, ligation was associated with significantly higher complication rates of extremity compartment syndrome.^[17,18]^ Considering the local economy and health conditions, repeated complications and treatment will increase the burden on patients. Recently, many scholars have proposed a strategy that combines resection and reconstruction to improve the resection rate and curative effect of surgery in the condition of infringement on large blood vessels.^[18–21]^ Thus, the method of reconstruction and choice of alternative blood vessel allograft are important factors affecting the safety of surgery and the prognosis of patients.^[22]^ Due to the issue of alternative blood vessels, it is common to use artificial/autologous/allogeneic blood vessels.^[17,20,21,23,24]^ In our center, we performed revascularization using donor abdominal aorta to replace the invaded vena cava during liver transplantation. We believe that allogeneic blood vessels have the same tissue structure and function as the host, with matched diameters and good long-term patency.^[21,23]^ Moreover, the cryogenic cryopreservation technique is maturing. This technique can extend the scope of allogeneic vasculature^[25]^ and may become the main potential source of blood substitutes in the future. It also provides a choice of grafts for vascular reconstruction for tumor patients with existing vascular invasion, making it possible to expand the radical resection of tumors.^[20,26,27]^

The literature has shown that 3D visualization techniques can help shorten the operation time and reduce bleeding.^[7]^ However, it did not show these benefits in this case. We assumed that there may be 3 reasons. First, due to the wide range of lesion invasion, the resection line did not follow the standard liver segment or the liver lobe. The wound surface was large, and the oozing was extensive. Second, a history of abdominal surgery combined with lesion violations led to severe liver adjacent organ adhesion and increased bleeding. Third, resuscitation of the air embolism also prolonged the operation time.^[8]^

## Author contributions

All the authors took part in the treatment of this patient. Ning Li, Dongdong Lin and Tiezheng Wang were responsible for the writing and revision of the paper. Guangming Li, Zhi Fu, and Daming Gao were responsible for the literature review and editing of the figures. All authors have read and approved the final version submitted.

**Conceptualization:** Tiezheng Wang, Ning Li.

**Validation:** Zhi Fu, Daming Gao, Dongdong Lin.

**Writing – original draft:** Ning Li.

**Writing – review & editing:** Zhi Fu, Daming Gao, Dongdong Lin.
